# International Epidemiology of Intracerebral Hemorrhage

**DOI:** 10.1007/s11883-012-0252-1

**Published:** 2012-04-27

**Authors:** M. Arfan Ikram, Renske G. Wieberdink, Peter J. Koudstaal

**Affiliations:** 1 Department of Epidemiology, Erasmus MC University Medical Center, P.O. Box 2040, 3000 CA Rotterdam, The Netherlands; 2 Department of Radiology, Erasmus MC University Medical Center, P.O. Box 2040, 3000 CA Rotterdam, The Netherlands; 3 Department of Neurology, Erasmus MC University Medical Center, P.O. Box 2040, 3000 CA Rotterdam, The Netherlands

**Keywords:** Intracerebral hemorrhage, Risk factors, Epidemiology, Microbleeds, Incidence, Hypertension

## Abstract

Intracerebral hemorrhage is the second most common subtype of stroke. In recent decades our understanding of intracerebral hemorrhage has improved. New risk factors have been identified; more knowledge has been obtained on previously known risk factors; and new imaging techniques allow for in vivo assessment of preclinical markers of intracerebral hemorrhage. In this review the latest developments in research on intracerebral hemorrhage are highlighted from an epidemiologic point of view. Special focus is on frequency, etiologic factors and pre-clinical markers of intracerebral hemorrhage.

## Introduction

Stroke is the second leading cause of death worldwide, and one of the leading causes of disability [1, 2]. With increasing life expectancy the burden of stroke is likely to increase worldwide with middle and low income countries particularly affected [3, 4]. Intracerebral hemorrhage is the second most common subtype of stroke after ischemic stroke and accounts for approximately 10 % to 20 % of all strokes [5].

Intracerebral hemorrhage occurs when a blood vessel within the brain parenchyma ruptures. Intracerebral hemorrhage can occur as a complication of a pre-existing lesion, such as vascular malformation or tumor, which is then referred to as secondary intracerebral hemorrhage. Primary intracerebral hemorrhage refers to intracerebral hemorrhage in the absence of a single clear underlying lesion and is the most frequent type of intracerebral hemorrhage. This review focuses on primary intracerebral hemorrhage .

Over the last decades much progress has been made in understanding stroke, though most research has focused on ischemic stroke. Nevertheless, steady progress has also been made in unravelling the causes of intracerebral hemorrhage. In recent years new risk factors for intracerebral hemorrhage have emerged; our understanding of known risk factors has improved; newer imaging techniques have allowed for identification of putative pre-clinical markers of intracerebral hemorrhage; and better treatment options have allowed for longer survival among patients suffering from intracerebral hemorrhage. Epidemiologic studies have played an essential role in these developments.

This review provides an overview of these recent epidemiologic developments in understanding intracerebral hemorrhage. The focus is on frequency, risk factors, pre-clinical markers, and prognostic indicators of intracerebral hemorrhage. Finally, we make a few recommendations for future epidemiologic research on intracerebral hemorrhage.

## Frequency of Intracerebral Hemorrhage

Whereas the age-standardized frequency of all stroke has decreased in the past decades, this decrease has primarily been driven by ischemic stroke [5]. Data on temporal trends for intracerebral hemorrhage are conflicting with some studies reporting a decrease in intracerebral hemorrhage in the last two decades [6, 7], whereas other studies show stable numbers in the same time period [8, 9], and yet other studies show an increase [10, 11]. Given the temporal trends in risk factors for intracerebral hemorrhage, one explanation for the conflicting results across studies is that the number of intracerebral hemorrhages due to hypertension has decreased in time due to better treatment, but is offset by an increase in number of intracerebral hemorrhages related to warfarin use [12]. Combining all available incidence data, a large meta-analysis recently concluded that most likely the incidence of intracerebral hemorrhage has not changed between 1980 and 2006 [13••]. The overall incidence during this time period was 24.6 per 100.000 person-years (95 % confidence interval 19.7–30.7), ranging from 1.8 to 129.6 per 100.000 person-years across studies. Among all studies included in that meta-analysis the incidence of intracerebral hemorrhage in Asian populations was almost twice that in other ethnicities (i.e. Black, Indian, Hispanic, Maori, White). Moreover, the incidence was 15 % lower in women than men, though not statistically significant (95 % confidence interval from 39 % decrease to 18 % increase). Consistent across studies was the finding that the incidence of intracerebral hemorrhage increased strongly with age, with persons aged 85 years and over having an almost ten-fold increase in yearly risk of intracerebral hemorrhage compared to persons aged 45 to 54 years [13••].

## Risk Factors

Much of the epidemiologic research on intracerebral hemorrhage has concentrated on identifying risk factors for intracerebral hemorrhage. Not only do risk factors provide etiologic insights into disease, but in case of modifiable risk factors, these can also become targets for preventive or therapeutic strategies. Moreover, risk factors can be utilized for their predictive or diagnostic value. This latter notion also brings forth an important methodological consideration in epidemiologic studies on intracerebral hemorrhage. Clinically, intracerebral hemorrhage is usually subtyped according to location into lobar or deep intracerebral hemorrhage [14]. This distinction is based on the presumed underlying etiology with lobar intracerebral hemorrhage thought to be caused primarily by cerebral amyloid angiopathy, and deep intracerebral hemorrhage more related to hypertensive vasculopathy. Similarly, another distinction of intracerebral hemorrhage is based on whether or not the intracerebral hemorrhage is related to warfarin use [7, 10, 14]. Whereas such distinctions may be useful in a clinical setting for prognostic and therapeutic stratification, for etiologic research it is important to note that intracerebral hemorrhage is a complex disease with multiple underlying etiologies and risk factors. An intracerebral hemorrhage is not caused by a single risk factor, but is more likely to be a result of a complex interplay of multiple risk factors—though a single or few risk factors may have a bigger role than others. Therefore, any distinction of intracerebral hemorrhage based on a single presumed underlying risk factor may preclude identification of additional risk factors. Nevertheless, having said this, we also note that such distinctions are useful if the aim is to identify risk factors that act in concert with either cerebral amyloid angiopathy or hypertension.

### Hypertension

Abundant evidence, both from case–control studies and cohort studies, shows that hypertension is the single most important risk factor for intracerebral hemorrhage [15, 16••]. Most of the intracerebral hemorrhages due to hypertension are thought to occur deep in the brain parenchyma, although associations with lobar intracerebral hemorrhage have been reported [17]. A meta-analysis across 11 case–control studies revealed that hypertensives had an almost 3.5-fold increased risk of intracerebral hemorrhage compared with normotensives [15]. Another meta-analysis showed that self-reported hypertension or a measured blood pressure of >160/90 increased the risk of intracerebral hemorrhage more than ninefold [16••]. It should be noted that these risk estimates were not adjusted for potential confounding variables. Therefore, the true risk increase due to hypertension is likely to be smaller, but nevertheless still very substantial.

The increased risk associated with blood pressure does not seem to be restricted to clinical hypertension. Studies have shown that blood pressure increases within the normal range are also associated with a linear increase in the risk of intracerebral hemorrhage [18].

Given the strength of association, the high prevalence of hypertension, and the readily available treatment options, blood pressure control is considered the main option for prevention of intracerebral hemorrhage.

### Smoking

Studies have consistently shown tobacco use as a risk factor for intracerebral hemorrhage [19, 20], though the effect size is not as large as for hypertension. These studies almost exclusively focus on cigarette smoking, but there is no reason not to generalize these findings to other modes of tobacco use, such as pipes and cigars [21].

Studies have demonstrated a dose–response relationship with the number of cigarettes smoked and risk of intracerebral hemorrhage. Moreover, the effect of smoking extends to former smoking, albeit the risk is largest for current smokers. The reported relative risk of current smokers versus non-smokers lies between 1.3 and 1.5 [15, 16••].

### Alcohol

Because the definition of high alcohol intake differs across various studies, it has been difficult to provide summary measures in meta-analyses. Nevertheless, there is compelling evidence relating high alcohol intake with a higher risk of intracerebral hemorrhage [15, 16••, 19]. Presumed pathways for this association include platelet dysfunction, coagulation disturbances, or endothelial damage. It is noteworthy that whereas for ischemic stroke moderate alcohol intake has been shown to be protective, the risk of intracerebral hemorrhage is increased suggesting a linear dose–response relationship between alcohol intake and intracerebral hemorrhage risk [19].

### Cholesterol and Lipids

Increasing evidence from longitudinal studies suggests that hypercholesterolemia is associated with a lower risk of intracerebral hemorrhage [22, 23]. This is in contrast to earlier case–control studies that implicated high cholesterol as risk factor for intracerebral hemorrhage [24]. The exact mechanism is still unclear but low cholesterol is thought to weaken the endothelial wall. More recent studies have investigated not only cholesterol, but several lipid fractions. These studies suggest that the associations with lipids are mainly driven by low triglyceride levels [25•]. How triglyceride levels lead to intracerebral hemorrhage still remains unclear.

### Diabetes Mellitus

Diabetes mellitus is of increasing interest as risk factor for intracerebral hemorrhage given the potential for relatively easy intervention and prevention strategies. A large meta-analysis of 102 prospective studies, including 698.782 participants, recently provided convincing evidence for diabetes as risk factor for intracerebral hemorrhage [26]. The reported relative risk was 1.6 (95 % confidence interval from 1.2 to 2.1) for persons with diabetes compared to persons without diabetes. There is also evidence from other studies implicating diabetes mellitus as risk factor for intracerebral hemorrhage [15].

### Cerebral Amyloid Angiopathy

After hypertension, the single most important risk factor for intracerebral hemorrhage is thought to be cerebral amyloid angiopathy. Cerebral amyloid angiopathy refers to the accumulation of β-amyloid in the media and adventitia of mostly cortical vessels, which can lead to leakage of blood through the vessel wall [27••]. Given the distribution of cerebral amyloid angiopathy, these intracerebral hemorrhage usually—though not exclusively—occur in the cortical brain regions, referred to as lobar intracerebral hemorrhage. The frequency of cerebral amyloid angiopathy is thought to increase with age, with almost half of all persons older than 90 showing some signs of cerebral amyloid angiopathy. Current estimates show that almost 50 % of intracerebral hemorrhages in a lobar region are related to amyloid angiopathy [28]. Although this percentage is high for a single risk factor, it is important to keep in mind that the remaining 50 % are caused by other risk factors, many of which are still unidentified. Apart from intracerebral hemorrhage, cerebral amyloid angiopathy also increases the risk of Alzheimer’s disease, suggesting partly overlapping mechanisms between intracerebral hemorrhage and Alzheimer’s disease [27••].

Most research on cerebral amyloid angiopathy currently focuses on its early identification and risk stratification based on any such early markers (see section on pre-clinical imaging markers). However, the causes of cerebral amyloid angiopathy are still unknown. Apart from genetic influences no clear modifiable risk factors have been identified to date (see section on genetic risk factors). Therefore, primary prevention opportunities related to cerebral amyloid angiopathy as risk factor for intracerebral hemorrhage are limited.

### Medication

Anticoagulant use, especially warfarin, is considered a risk factor of intracerebral hemorrhage. Evidence for this association comes from various sources [7, 10]. It is important to note, however, that benefits of warfarin far outweigh the increased risk of intracerebral hemorrhage associated with it. Still, this demonstrates the urgent need for better risk stratification strategies in order to better balance the benefits and adverse events associated with warfarin use. Given that warfarin use has rapidly increased in the last decades, studies have also shown a drastic increase in intracerebral hemorrhage related to warfarin use [10], though this notion is contested by other studies [7]. The increased risk of intracerebral hemorrhage related with anticoagulants also extends to other coumarin derivatives, such as acenocoumarol and fenprocoumon, which are more often used than warfarin in European settings [29]. Data on other anticoagulants are scarce, though there is a suggestion of an increased risk of intracerebral hemorrhage related with direct thrombin inhibitors [30]. However, more data are needed before a more conclusive judgement can be made.

Likewise, a large meta-analysis showed that aspirin use increases the risk of intracerebral hemorrhage [31], though here too the benefits of aspirin far outweigh the potential risks associated with its use.

### Genetic Risk Factors

Evidence for a genetic predisposition for intracerebral hemorrhage comes from family studies. Several single-gene disorders leading to intracerebral hemorrhage have been identified, and include familial cerebral amyloid angiopathy due to mutations in the *APP* gene and cerebral autosomal dominant arteriopathy with subcortical infarcts and leukoencephalopathy (CADASIL) due to mutations in the *NOTCH3* gene [32]. However, these monogenetic disorders are rare and explain only a small proportion of the genetic predisposition of intracerebral hemorrhage.

Instead, the genetics of primary intracerebral hemorrhage are thought to be more complex with many other genes involved each with a small individual effect [32]. Because of their modest effect sizes, many of such genes are still unidentified. So far, only the *APOE* gene has been identified as a robust genetic risk factor for intracerebral hemorrhage. *APOE* is considered a strong risk factor for cerebral amyloid angiopathy and it is for a large part via this underlying etiology that *APOE* relates with intracerebral hemorrhage [27••]. However, *APOE* has also been shown to affect blood vessels via other mechanisms.

Apart from *APOE*, many other candidate genes have been implicated, although evidence for such genes has been limited. These genes include *ACE*, *APOH*, *Factor VII* and *Factor XIII*, and *IL-6* [32]. The advent of genome-wide association studies, in which hundreds of thousands of genetic markers are tested concomitantly, provides a unique opportunity to identify additional genes with modest effects. The success and feasibility of genome-wide association studies has already been demonstrated in ischemic stroke and dementia [33, 34]. However, the necessity of having large sample sizes currently limits the full potential of genome-wide association studies for intracerebral hemorrhage. Nevertheless, *CR1*, a novel gene for Alzheimer’s disease identified using genome-wide association studies, has also been implicated in intracerebral hemorrhage [35].

### Emerging Risk Factors

Apart from the aforementioned risk factors, for which associations are robust and have been established, there are several other putative risk factors for which a solid body of evidence is emerging.

Regular physical activity has been suggested to be protective of intracerebral hemorrhage, though studies have not shown conclusive evidence [16••, 36]. Dietary factors have been implicated in intracerebral hemorrhage, although it is still unclear which dietary constituents drive this association [19, 37].

Emerging evidence further shows that poor kidney function is a novel risk factor for stroke, including intracerebral hemorrhage [38]. This risk is not only restricted to patients with overt kidney disease, but seemingly extends to the general population. However, it is worth noting that not all studies find associations between markers of kidney disease and intracerebral hemorrhage [39]. This shows the need for further studies on this relationship.

There is also increasing interest in body-mass index as risk factor for intracerebral hemorrhage [40], though it is likely not a direct cause but rather an indicator of an underlying process. Interestingly, both low and high body mass index increase the risk of intracerebral hemorrhage [40].

## Pre-Clinical Imaging Markers

Given the potential public health impact of early identification of persons at high-risk of intracerebral hemorrhage, many epidemiological studies have focused on identifying pre-clinical non-invasive imaging markers for intracerebral hemorrhage. We focus here on two promising markers that are related to magnetic resonance imaging (MRI) of the brain and retinal imaging.

### Cerebral Microbleeds on Magnetic Resonance Imaging

In recent years, newer MRI techniques have led to the discovery of cerebral microbleeds [41•]. These are small loci of chronic blood products in brain tissue, caused by leakage of blood vessels. Studies have shown a high prevalence and incidence of these lesions, both in a clinical setting and in the general elderly population [42, 43]. Given the presumed overlapping etiology, microbleeds are of interest as putative precursors of intracerebral hemorrhage. In line with this, microbleeds are also categorized into deep and lobar according to the presumed underlying cause: there are compelling data relating lobar microbleeds to cerebral amyloid angiopathy and deep microbleeds to hypertensive vasculopathy [41•]. Recent studies have also found similarities between intracerebral hemorrhage and microbleeds with regard to shared risk factors, i.e. *APOE*, hypertension, and lipid profile [25•, 44]. However, not only is it of interest to investigate shared risk factors, but also to focus on non-overlapping risk factors and to assess to what extent these point towards a different pathology underlying microbleeds and intracerebral hemorrhage.

With regards to clinical outcomes of cerebral microbleeds, several clinical studies among stroke patients have related microbleeds to the risk of (recurrent) intracerebral hemorrhage [27••]. Moreover, given the overlap between cerebral amyloid angiopathy and Alzheimer pathology, microbleeds have also been associated with Alzheimer’s disease [45].

Still, to fully establish cerebral microbleeds as precursor (and thus predictor) of intracerebral hemorrhage population-based longitudinal studies are needed, which are currently lacking. In coming years, the exact role of microbleeds as putative pre-clinical marker of intracerebral hemorrhage is set to be established.

### Retinal Imaging

The retinal vasculature shares many morphologic and physiologic properties with intracerebral vasculature. Because the retinal vasculature is easily accessible in vivo using retinal imaging, there is an increasing interest to use the retina as model to study pathologic changes in the cerebral vasculature. Retinal signs that have been associated with intracerebral hemorrhage include retinal vessel calibres, signs of age-related macular disease, retinopathy, focal narrowing of arterioles, and arteriovenous nicking [46–48]. However, many of these signs are not specific for intracerebral hemorrhage and have also been associated with ischemic stroke and its subtypes. Therefore, despite convincing associations, the predictive accuracy of retinal imaging for risk of intracerebral hemorrhage is yet to be determined.

## Prognosis after Intracerebral Hemorrhage

Despite aggressive and newer management strategies, the prognosis of patients with intracerebral hemorrhage remains very poor: case-fatality at 1 month is over 40 % and has not improved in the last decades [13••]. Similarly, of those who survive only a small proportion reaches independent life after 1 year, with estimates varying between 12 % and 39 % [13••]. These numbers show that intracerebral hemorrhage is not only a very lethal disease, but that remaining survivors pose a significant burden on health care resources.

It is worth noting that most studies investigating prognosis of intracerebral hemorrhage do so until 1 year after intracerebral hemorrhage [20, 49]. Very few studies have investigated long-term prognosis of intracerebral hemorrhage beyond 3 years [50], usually because people are discharged from clinical follow-up. It remains to be seen therefore, whether the adverse prognosis after intracerebral hemorrhage remains poor or stabilizes in the long-term. Population-based studies would provide an ideal setting to study the long-term prognosis after intracerebral hemorrhage, given their better follow-up of patients that discontinue clinical evaluations. In this light, it would be equally interesting to investigate determinants of long-term prognosis after intracerebral hemorrhage.

## Conclusions

Intracerebral hemorrhage is a multi-factorial disease caused by several interacting and overlapping risk factors and etiologies. Hypertension remains the most important risk factor followed by cerebral amyloid angiopathy. However, the increased use of anticoagulants poses new challenges to better prevent intracerebral hemorrhage as complication. Emerging risk factors have also been identified providing new pathways for further research. New imaging techniques will in coming years be assessed for their ability to identify persons at an increased risk of intracerebral hemorrhage, which will substantially improve pre-clinical risk-stratification.

However, despite these advances, intracerebral hemorrhage still poses a substantial burden on healthcare systems. More worryingly, over the last decades the incidence and prognosis of intracerebral hemorrhage have remained stable. This indicates that there is still a long way to go before we have a full understanding of intracerebral hemorrhage. Epidemiologic research will continue to be one of the cornerstones in research on intracerebral hemorrhage.
